# Angiotensin II receptor blocker as a novel therapy in acute lung injury induced by avian influenza A H5N1 virus infection in mouse

**DOI:** 10.1007/s11427-015-4814-7

**Published:** 2015-02-07

**Authors:** YiWu Yan, Qiang Liu, Ning Li, JianChao Du, Xiao Li, Chang Li, NingYi Jin, ChengYu Jiang

**Affiliations:** 1 State Key Laboratory of Medical Molecular Biology, Institute of Basic Medical Sciences, Chinese Academy of Medical Sciences; Department of Biochemistry and Molecular Biology, Peking Union Medical College, Tsinghua University, Beijing, 100005 China; 2 Genetic Engineering Laboratory, Institute of Military Veterinary, Academy of Military Medical Sciences, Changchun, 130062 China; 3 State Key Laboratory of Biotherapy/Collaborative Innovation Center for Biotherapy, West China Hospital, Sichuan University, Chengdu, 610000 China

**Keywords:** Influenza, Acute Lung Injury, Losartan, Avian Influenza, H5N1 Virus

YAN YiWu[1†], LIU Qiang[1†], LI Ning[1], DU JianChao[1], LI Xiao[2], LI Chang[2], JIN NingYi[2*] & JIANG ChengYu[1,3*]

Dear Editor,

Since the first recognized case of human infection with avian influenza H5N1 virus in 1997, the worldwide spread and re-emergence of this highly pathogenic influenza virus have led to 694 human infection cases. Of these cases, 402 died from 2003 to 2015 (http://www.who.int/influenza/human_animal_interface/EN_GIP_20150106Cumulative-NumberH5N1cases.pdf?ua=1), with a morbidity rate of 57.9%, which is much higher than that of seasonal influenza H1N1 virus. The outbreak of the H7N9 virus epidemic in 2013 has been a warning sign that the occurrence of new influenza pandemics and the sustained circulation of these viruses in poultry and other animals pose a persistent threat to public health. In addition, recent studies on influenza virus transmission proved that only a few amino acid mutations are required to make H5N1 virus transmissible among ferrets, an animal model that resembles human response to influenza virus infection [1,2]. Considering the potential of mutated avian influenza virus being both easily transmitted and highly pathogenic to human, it is time to take measures before the next coming influenza pandemic.

After entering its host cells, influenza virus first massively replicates, and then it induces acute and severe immune responses, which lead to serious respiratory injuries and consequently acute lung injury (ALI) or acute respiratory distress syndrome (ARDS) [3]. At present, due to limited therapeutic strategies for influenza virus infection or ARDS, the mortality rate for humans infected with high pathogenic H5N1 remains high. Therefore, to find effective prophylactic or therapeutic agents that potentially alleviate H5N1 virus-induced lung injury should be of great importance.

Re-investigating existing drugs is an economical and time-saving approach to satisfy unmet medical needs because the safety, pharmacology, formulation and potential toxicity of shelved drugs have been largely established. Our previous study has shown that angiotensin II receptor blocker (ARB) losartan could effectively reduce ALI induced by SARS-CoV [4]. We recently reported that avian influenza A H5N1 virus infection disrupted host renin-angiotensin system (RAS) in mice and human plasma level of angiotensin II was markedly elevated in both H5N1 and H7N9 infected patients [5,6]. Consisting with that, we found administration of recombinant human ACE2 protein effectively ameliorated the murine ALI induced by H5N1 virus infection. Dysregulated angiotensin II level is highly associated with diseases such as hypertension and cardiac

†Contributed equally to this work
*Corresponding author (email: jiang@pumc.edu.cn; ningyik@126.com)

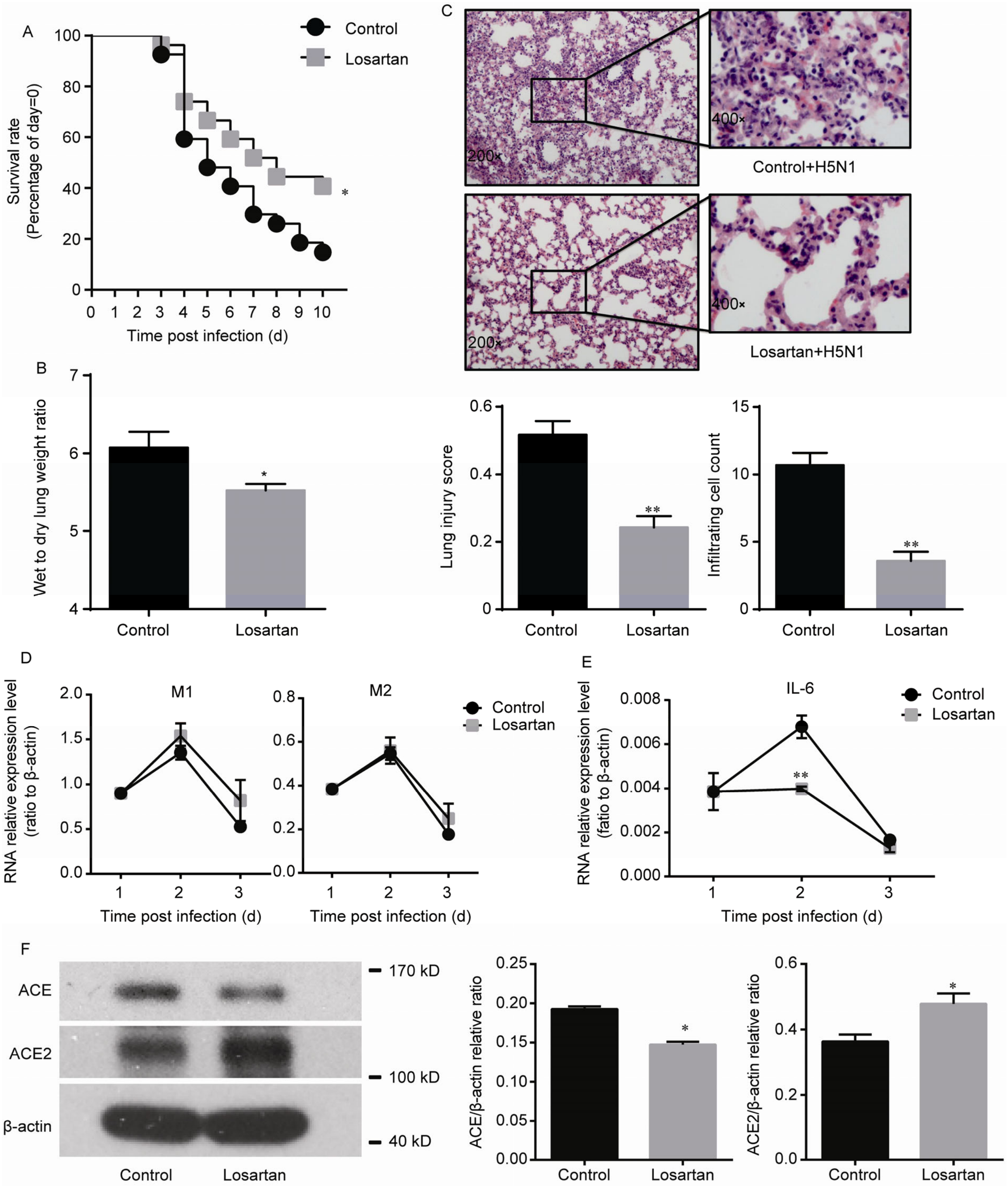

**Figure 1** Losartan can effectively ameliorate ALI induced by avian influenza A H5N1 virus infection in mice. A, Survival rate of C57/B6 mice receiving losartan or vehicle control once per day for continuously 4 days after intratracheal instillation of H5N1 virus. Survival rate data were collected from 27 mice per group. B, Wet/dry weight ratio, indicating the severity of lung edema, in H5N1-infected mice with or without losartan treatment. Lung tissues were obtained on Day 7 after virus instillation. Data were from 6-7 mice per group. C, Representative lung histopathology of C57/B6 mice on Day 4 after virus infection following therapeutic treatment with losartan (lower panels) compared to non-treated group (upper panels). The original magnification was 200× (left panels) followed by an increased magnification (400×, right panels). Totally 100 fields were analyzed with 6 mice in each group. The bar graph stands for lung injury scores (left) and the number of infiltrating cells in infected lung tissues (right). Data were presented as mean±SEM. D, RT-qPCR analysis of M1 and M2 components of H5N1 virus. Data were presented as relative expression level of M1 and M2 to β-actin 3–4 mice per group. E, RT-qPCR analysis of IL-6 expression relative to β-actin in mouse lung tissues following therapeutic treatment with losartan or vehicle control. Data from 3–4 mice per group were analyzed. F, Western blot analysis of ACE and ACE2 expression in mouse lung tissues following losartan treatment, with β-actin as internal control. Representative blots were shown (left), and a pool of four mice's lung lysates was analyzed, presented as bar graphs and shown in the middle (for ACE) and on the right (for ACE2). Data were presented as mean of ACE- or ACE2-to-β-actin ratio±SEM from 4 mice in each group. *, $P \leqslant 0.05$; **, $P \leqslant 0.01$.

disease [7] and two classes of drugs have already been developed and clinically applied to maintain the normal function of RAS. The first class is ACE inhibitors (ACEis), which inhibit ACE from cleaving angiotensin I to generate angiotensin II. The second class is angiotensin II receptor blockers (ARBs), which block the interaction between angiotensin II and its type I receptor (AT1R) to suppress the downstream immune responses [8]. We thus hypothesized that these drugs, which demonstrated the same therapeutic consequence as increased ACE2 protein expression, may also be effective for the treatment of H5N1 infections.

Previous studies have shown that ARBs perform better than ACEi in respiratory disorders such as ARDS induced by bacterial infection, as angiotensin II can be synthesized by some alternative enzymes such as chymase, cathepsin and trypsin [9,10]. Thus in our study, we focused on losartan, the first orally active non-peptide selective ARB, to further investigate its therapeutic role in ALI induced by H5N1 virus.

To test the potential therapeutic effect of losartan on ALI induced by live H5N1 virus, we used Losartan developed by DuPont Merck Pharmaceutical Co. under the code DuP753 or MK954, which is a potassium salt of 2-n-butyl-4-chloro-5-hydroxymethyl-1-{(2′-[1H-tetrazol-5-yl]biphenyl-4-yl)methyl}imidazole [11], with a molecular mass of 461 Da. We found that administration of losartan at a dose clinically equivalent to human significantly improved the survival rate in H5N1 virus-infected mice (Figure 1A). Then, the severity of lung injury was determined by lung histopathology and the wet/dry lung weight ratio, which is an indicator of lung edema. Mice treated with losartan showed alleviated lung edema and improved lung histopathology, demonstrated as lowered lung injury scores and a reduced number of infiltrating leukocytes (Figure 1B and C). Taken together, these results suggest that losartan, a conventional anti-hypertension medication, is a promising strategy to ameliorate ALI and improve survival of mice infected with live avian influenza A H5N1 virus.

Though effective when treated with Losartan, the viral load in the lung tissue of infected mice was not reduced after losartan treatment, indicating that losartan may not influence H5N1 viral entry or replication (Figure 1D). Since the binding of angiotensin II with AT1R promotes inflammation and vascular permeability of lung tissue [12], which at least partially accounts for ALI induced by H5N1 virus, we supposed that the effect of losartan may result from a prevention of excessive downstream inflammatory responses. In agreement with that, IL-6, one of the primary contributor cytokines downstream of AT1R, was also significantly down-regulated in losartan-treated group (Figure 1E). Furthermore, a remarkable recovery of ACE2 expression and down-regulation of ACE in lung tissues of losartan-treated mice were also observed 48 h after H5N1 virus infection (Figure 1F). These data support the above notion that the mechanism of action of losartan in the context of H5N1 influenza virus infection is mediated *via* RAS pathway.

Dr. David Fedson has recently called for the use of statin, ARB, and ACEi in next influenza pandemic [13], and previous population-based study has clearly demonstrated that ARB inpatient application for severe pneumonia could significantly reduce 30-day mortality rate in hospital [14]. Here, we further provided experimental evidence that losartan and perhaps other ARBs should be considered as candidate therapies for clinical treatment of H5N1-infected patients. Herein randomized clinical trials on ARB treatment for ARDS are indeed in need.

*This work was supported by the Ministry of Science and Technology of China (2015CB553400/6), National Natural Science Foundation of China (81230002 and 81490531).*

1 Imai M, Watanabe T, Hatta M, Das SC, Ozawa M, Shinya K, Zhong G, Hanson A, Katsura H, Watanabe S, Li C, Kawakami E, Yamada S, Kiso M, Suzuki Y, Maher EA, Neumann G, Kawaoka Y. Experimental adaptation of an influenza H5 HA confers respiratory droplet transmission to a reassortant H5 HA/H1N1 virus in ferrets. Nature, 2012, 486: 420–428
2 Herfst S, Schrauwen EJ, Linster M, Chutinimitkul S, de Wit E, Munster VJ, Sorrell EM, Bestebroer TM, Burke DF, Smith DJ, Rimmelzwaan GF, Osterhaus AD, Fouchier RA. Airborne transmission of influenza A/H5N1 virus between ferrets. Science, 2012, 336: 1534–1541
3 Peiris JS, Cheung CY, Leung CY, Nicholls JM. Innate immune responses to influenza A H5N1: friend or foe? Trends Immunol, 2009, 30: 574–584
4 Kuba K, Imai Y, Rao S, Gao H, Guo F, Guan B, Huan Y, Yang P, Zhang Y, Deng W, Bao L, Zhang B, Liu G, Wang Z, Chappell M, Liu Y, Zheng D, Leibbrandt A, Wada T, Slutsky AS, Liu D, Qin C, Jiang C, Penninger JM. A crucial role of angiotensin converting enzyme 2 (ACE2) in sars coronavirus-induced lung injury. Nat Med, 2005, 11: 875–879
5 Zou Z, Yan Y, Shu Y, Gao R, Sun Y, Li X, Ju X, Liang Z, Liu Q, Zhao Y, Guo F, Bai T, Han Z, Zhu J, Zhou H, Huang F, Li C, Lu H, Li N, Li D, Jin N, Penninger JM, Jiang C. Angiotensin-converting enzyme 2 protects from lethal avian influenza A H5N1 infections. Nat Commun, 2014, 5: 3594
6 Huang F, Guo J, Zou Z, Liu J, Cao B, Zhang S, Li H, Wang W, Sheng M, Liu S, Pan J, Bao C, Zeng M, Xiao H, Qian G, Hu X, Chen Y, Chen Y, Zhao Y, Liu Q, Zhou H, Zhu J, Gao H, Yang S, Liu X, Zheng S, Yang J, Diao H, Cao H, Wu Y, Zhao M, Tan S, Guo D, Zhao X, Ye Y, Wu W, Xu Y, Penninger JM, Li D, Gao GF, Jiang C, Li L. Angiotensin II plasma levels are linked to disease severity and predict fatal outcomes in H7N9-infected patients. Nat Commun, 2014, 5: 3595
7 Kim S, Iwao H. Molecular and cellular mechanisms of angiotensin II-mediated cardiovascular and renal diseases. Pharmacol Rev, 2000, 52: 11–34
8 Robles NR, Cerezo I, Hernandez-Gallego R. Renin-angiotensin system blocking drugs. J Cardiovasc Pharmacol Ther, 2014, 19: 14–33
9 Khan MG. Cardiac Drug Therapy. Springer, 2007
10 Raiden S, Nahmod K, Nahmod V, Semeniuk G, Pereira Y, Alvarez C, Giordano M, Geffner JR. Nonpeptide antagonists of AT1 receptor for angiotensin II delay the onset of acute respiratory distress syndrome. J Pharmacol Exp Ther, 2002, 303: 45–51
11 Inada Y, Wada T, Shibouta Y, Ojima M, Sanada T, Ohtsuki K, Itoh K, Kubo K, Kohara Y, Naka T, et al. Antihypertensive effects of a highly potent and long-acting angiotensin II subtype-1 receptor antagonist, (+-)-1-(cyclohexyloxycarbonyloxy)ethyl 2-ethoxy-1-[[2′-(1h-tetrazol-5-yl)biphenyl-4-yl]methyl]-1h-benzimidazole-7-carboxylate (TCV-116), in various hypertensive rats. J Pharmacol Exp Ther, 1994, 268: 1540–1547

12 Imai Y, Kuba K, Penninger JM. The discovery of angiotensin-converting enzyme 2 and its role in acute lung injury in mice. Exp Physiol, 2008, 93: 543–548

13 Fedson DS. How will physicians respond to the next influenza pandemic? Clin Infect Dis, 2014, 58: 233–237

14 Mortensen EM, Nakashima B, Cornell J, Copeland LA, Pugh MJ, Anzueto A, Good C, Restrepo MI, Downs JR, Frei CR, Fine MJ. Population-based study of statins, angiotensin II receptor blockers, and angiotensin- converting enzyme inhibitors on pneumonia-related outcomes. Clin Infect Dis, 2012, 55: 1466–1473

**Open Access** This article is distributed under the terms of the Creative Commons Attribution License which permits any use, distribution, and reproduction in any medium, provided the original author(s) and source are credited.

## Supporting Information

### Supplementary materials

The supporting information is available online at life.scichina.com and link.springer.com. The supporting materials are published as submitted, without typesetting or editing. The responsibility for scientific accuracy and content remains entirely with the authors.

## Electronic supplementary material

Supplementary material, approximately 356 KB.
