# Supplementary material for: Angiotensin II receptor blocker as a novel therapy in acute lung injury induced by avian influenza A H5N1 virus infection in mouse
Source: Sci China Life Sci. 2015 Feb 7;58(2):208–11. doi: 10.1007/s11427-015-4814-7 (PMC7088914; doi:10.1007/s11427-015-4814-7)
Supplement: Supplementary file 1 — Supplementary material, approximately 356 KB. [file 11427_2015_4814_MOESM1_ESM.pdf]

SCIENCE CHINA
Life Sciences

Supporting Information

February 2015 Vol.58 No.2: 1–2
doi: 10.1007/s11427-015-4814-7

1 Virus

The influenza virus used in this study was H5N1 (A/Jilin/9/2004(H5N1)). Live virus experiments were performed in Biosafety Level 3 facilities under governmental and institutional guidelines. Viruses were propagated by inoculation into 9- to 11-day-old SPF embryonated fowl eggs via the allantoic route. Hemagglutinating allantoic fluid was collected from eggs and used directly.

2 Reagents

For these experiments, losartan were purchased from Sigma-Aldrich (Saint Louis, USA). The ACE monoclonal antibody was purchased from Cell Signaling Technology (Massachusetts, USA), the ACE2 monoclonal antibody was purchased from Epitomics (California, USA) and β-actin monoclonal antibody was purchased from Sigma-Aldrich (Saint Louis, USA). Primers used in this study were synthesized by Invitrogen (California, USA).

3 Animal handling

Animal experiments were conducted in the Institute of Military Veterinary Medicine, Academy of Military Medical Sciences, in accordance with governmental and institutional guidelines. Four-week-old male C57/B6 mice were purchased from Vital River Laboratories (Beijing, China). Mice were caged in a specific pathogen-free facility. Lung injury was induced in all groups except for the pulmonary edema testing through intratracheal injection of vehicle control or virus (10^6 $TCID_{50}$) using a technology that we have previously described for severe acute respiratory syndrome (SARS) spike protein and acid-induced lung injury [4]. Mice for the use of pulmonary edema testing were treated with intratracheal injection of vehicle control or virus (5×10^5 $TCID_{50}$). Losartan (50 mg kg^{-1}) was injected i.v. once per day for continuously 4 days after H5N1 virus instillation.

4 Survival rate

Mice were treated as described above and survival rate of each group (n=27) was recorded consecutively for 10 days. Survival rate was analyzed by Kaplan-Meier survival analysis.

5 Lung tissue slides for histopathological examination

Four days after the instillation of H5N1 virus, the mice were euthanized. The lungs were dislodged from the thoracic cavity and placed in a glass vial containing approximately 50 mL of fixative. Each glass vial was assigned a number unknown (with respect to treatment) to the pathologists. The lungs were fixed for at least 48 h before further processing. The formalin-fixed mouse lungs were embedded in paraffin, thin-sectioned coronally, and mounted onto glass microscope slides using standard histopathological techniques. The sections were stained with hematoxylin-eosin. For each group, six mice were euthanized, and the mouse lung tissues were examined under the 200× and 400× objectives. For each mouse, a slide containing one lung section was examined independently by three pathologists blinded to the treatment or genotype. The numbers of inflammatory cells were counted in 100 microscopic fields, and lung injury scores were quantified inclusive of the alveolar wall thickness, hyaline membranes, and proteinaceous debris filling the airspaces to yield an overall score between 0 and 1.

© The Author(s) 2015. This article is published with open access at link.springer.com

6 Assessment of pulmonary edema

Mice were treated as described above. Lung injury was induced in mice through intratracheal injection of vehicle control or virus (5×10^5 $TCID_{50}$). Lungs were assessed for their wet weight 7 days after virus injection and dried in a 65°C oven for 24 h to obtain the dry weight.

7 Western blot analysis

Mice were intratracheal instillated of H5N1 virus (10^6 $TCID_{50}$) and the lung tissues were collected at the indicated time points. Lung tissues were homogenized in ice-cold lysis buffer (50 mmol L^{-1} tris-HCl (pH 7.5), 150 mmol L^{-1} NaCl, 1.0% Triton X-100, 20 mmol L^{-1} EDTA, 1 mmol L^{-1} Na_3VO_4, 1 mmol L^{-1} NaF, and protease inhibitors). Tissue lysates were resolved by SDS-polyacrylamide gel electrophoresis (SDS-PAGE), and proteins were transferred onto a nitrocellulose filter membrane. Membranes were incubated with the appropriate primary antibodies and then with HRP-conjugated secondary antibodies. Binding of secondary antibody was visualized with the Kodak film exposure detection system, and the film was scanned and analyzed.

8 Real-time quantitative PCR analysis

Mice were intratracheal instillated of H5N1 virus (10^6 $TCID_{50}$) and the lung tissue were collected at the indicated time points. Total RNA were isolated with Trizol reagent (Invitrogen). Complementary DNA (cDNA) was synthesized from 1.0 μg of total RNA with the High Capacity cDNA Reverse Transcription kit (Applied Biosystems). PCR amplification assays were performed with the LightCycler 480 SYBR Green I Master (Roche Applied Science, Cat. No. 04707516001, USA) on an LightCycler 480 PCR System. Samples were normalized based on the expression the gene encoding mice beta-actin as a reference. The specific primers used were as follows: *M1* forward: 5′-CTCTCTATCATCCCGTCAG-3′; *M1* reverse: 5′-GTCTTGTCTTTAGCCATTCC-3′; *M2* forward: 5′-ATTGTGGATTCTTGATCGTC-3′; *M2* reverse: 5′-TGACAAAAT-GACCATCGTC-3′; *IL-6* forward: 5′-CCGCTATGAAGTTCCTCT-3′; *IL-6* reverse: 5′-CTCTGTGAAGTCTCCTCTC-3′; mouse *beta-actin* forward: 5′-CTCTCCCTCACGCCATCC-3′; mouse *beta-actin* reverse: 5′-CGCACGATTTCCCTCTCAG-3′.

9 Statistical analysis

All of the data are shown as the mean±SEM. Measurements at single time points were analyzed by analysis of variance (ANOVA), and if they demonstrated significance, the measurements were further analyzed by a two-tailed *t*-test and $P\leqslant0.05$ is considered statistically significant. Survival data were analyzed by Kaplan-Meier survival analysis. All of the statistical tests were conducted using GraphPad Prism 5.0 (GraphPad Software, San Diego, CA, USA).
